# Local Chemical Environment Governs Anode Processes in CO_2_ Electrolyzers

**DOI:** 10.1021/acsenergylett.1c01937

**Published:** 2021-10-07

**Authors:** Ádám Vass, Balázs Endrődi, Gergely Ferenc Samu, Ádám Balog, Attila Kormányos, Serhiy Cherevko, Csaba Janáky

**Affiliations:** † Department of Physical Chemistry and Materials Science, Interdisciplinary Excellence Centre, University of Szeged, Aradi Square 1, Szeged H-6720, Hungary; ‡ Helmholtz-Institute Erlangen-Nürnberg for Renewable Energy (IEK-11), Forschungszentrum Jülich GmbH, Egerlandstraße 3, 91058 Erlangen, Germany

## Abstract

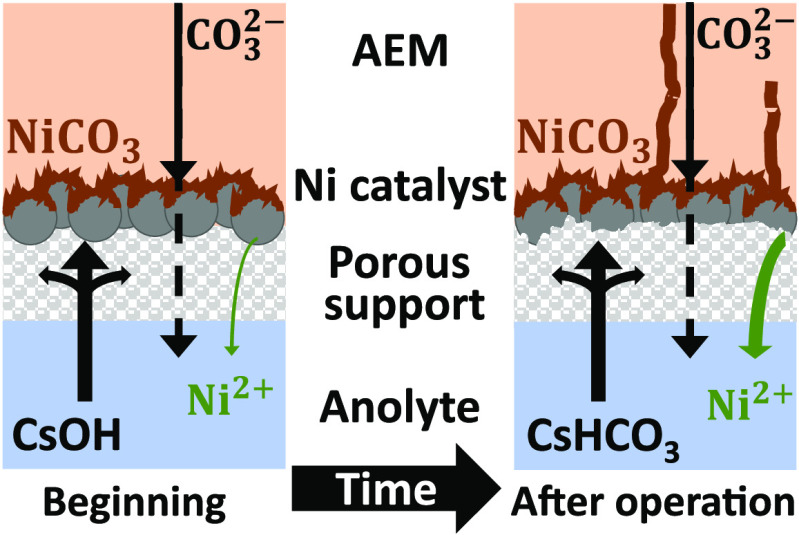

A major goal within the CO_2_ electrolysis community is to replace the generally used Ir anode catalyst with a more abundant material, which is stable and active for water oxidation under process conditions. Ni is widely applied in alkaline water electrolysis, and it has been considered as a potential anode catalyst in CO_2_ electrolysis. Here we compare the operation of electrolyzer cells with Ir and Ni anodes and demonstrate that, while Ir is stable under process conditions, the degradation of Ni leads to a rapid cell failure. This is caused by two parallel mechanisms: (i) a pH decrease of the anolyte to a near neutral value and (ii) the local chemical environment developing at the anode (i.e., high carbonate concentration). The latter is detrimental for zero-gap electrolyzer cells only, but the first mechanism is universal, occurring in any kind of CO_2_ electrolyzer after prolonged operation with recirculated anolyte.

Iridium is almost exclusively used as the anode catalyst in polyelectrolyte membrane water electrolyzers.^1^ Based on the similarities of water and CO_2_ electrolysis (i.e., the anode reaction is the oxygen evolution reaction (OER) in both cases), Ir quickly became the preferred anode catalyst for laboratory-scale experiments on the electrochemical CO_2_ reduction reaction (CO2RR).^2^ During CO_2_ electrolysis, an alkaline electrolyte solution (anolyte) is typically recirculated in the anode compartment to ensure a high reaction rate.^3^ Based on the slow but continuous anodic dissolution of Ir in alkaline media,^4^ scientists often claim that it must be replaced in alkaline CO_2_ electrolyzers.^5,6^

The catalyst replacing Ir must possess high OER activity and stability under operational conditions. Furthermore, it should meet a number of practical requirements, such as low price, high electrochemically active surface area, and high conductivity.^7^ Notable research efforts have been devoted to explore the OER activity of different transition metals and their compounds, including Ni, Co, and Fe oxides in alkaline water electrolysis.^8^ Directly translating this knowledge to CO2RR, however, is not straightforward, because of the considerably different operation conditions. Furthermore, different electrolyzer cell architectures (e.g., microfluidic vs zero-gap) might provide different chemical environments at the anode. At first glance, the anode process of CO2RR employing basic anolytes is alkaline water oxidation. Taking a look at the Pourbaix diagrams,^9^ Ni seems an ideal choice as anode catalyst for CO2RR, as it is stable at high pH values even at high positive potentials. Furthermore, the market price of Ni is about 10 000 times lower than that of Ir.^10^ Replacing Ir with Ni would therefore mean a substantial cost reduction in CO_2_ electrolyzer cells,^11^ which could strongly support the industrialization of this technology.^12^ All these factors together make Ni a viable candidate to be compared with the benchmark Ir, exploring the factors affecting the anode performance in CO2RR.

Ni foil, mesh, and foam modified by various methods like etching,^13^ laser ablation,^14,15^ transition metal electrodeposition,^16−19^ and spray coating of nanoparticles,^20−23^ are commonly investigated as OER electrodes. Ni has already been employed as anode catalyst in recent studies on CO2RR.^24−32^ Concentrated electrolyte solutions (e.g., 1 M KOH) were used in most of these studies, and stability was mostly demonstrated in electrolyzer cells operating with liquid catholyte for relatively short time periods. High electrolyte concentration, however, leads to the formation of alkali metal carbonates/bicarbonates in the gas diffusion electrode (GDE) cathode, leading to the clogging of the gas channels and the concurrent selectivity decrease for CO2RR (with the simultaneous increase of the hydrogen evolution reaction (HER) rate).^33−36^ Ni foam has also been applied in non-zero-gap flow cells with a bipolar membrane or paired with a molecular catalyst for CO2RR.^26,27,31^ Additionally, NiO nanoparticles were employed to oxidize 5-(hydroxymethyl)furfural to 2,5-furandicarboxylic acid, paired with CO2RR.^30^ In another example, NiFe foam was anodized in 0.1 M KHCO_3_ to make NiFe hydroxide carbonate, which was used as OER catalyst paired with cobalt phthalocyanine/carbon nanotube CO2RR catalyst.^28^ Ni foam and stainless steel fiber felt were tested in OER under alkaline (1 M KOH, pH = 14) and neutral (1 M potassium phosphate buffer, pH = 7) conditions, and Ni foam was highly unstable compared to stainless steel under neutral conditions.^37^

In anion exchange membrane (AEM)-separated CO_2_ electrolyzer cells, carbonate ions (migrating from the cathode to the anode) maintain the ion conductance, especially at high current density.^3,38,39^ If the anolyte is recirculated (typical scenario), the continuous carbonate transport decreases its bulk pH.^40^ In the case of zero-gap electrolyzer cells, the carbonate ion flux directly reaches the anode catalyst layer (i.e., it is not diluted by a liquid electrolyte, unlike in microfluidic cells), causing a high carbonate ion concentration. In contrast to water electrolyzer cells, where OH^–^ ions are the charge carriers between the electrodes, the anodically forming H^+^ ions are not neutralized instantly, which leads to an acidic surface pH.^40^ This also means that the local pH at the anode catalyst surface is lower than the bulk solution pH.

The CO_2_ electroreduction community has paid limited attention to the anode catalysts and reactions so far. In this study, our aim was three-fold: (i) to investigate and explain the reason behind the experimental findings that Ir is a stable OER catalyst in zero-gap alkaline electrolyzer cells, (ii) to define the requirements of an OER catalyst for long-term CO_2_ electrolysis, and (iii) to scrutinize whether Ir can be replaced by Ni as anode catalyst.

## Comparing the Operation of a Zero-Gap CO_2_ Electrolyzer Cell Using Ir and Ni Anode Catalysts

**Scheme 1:**
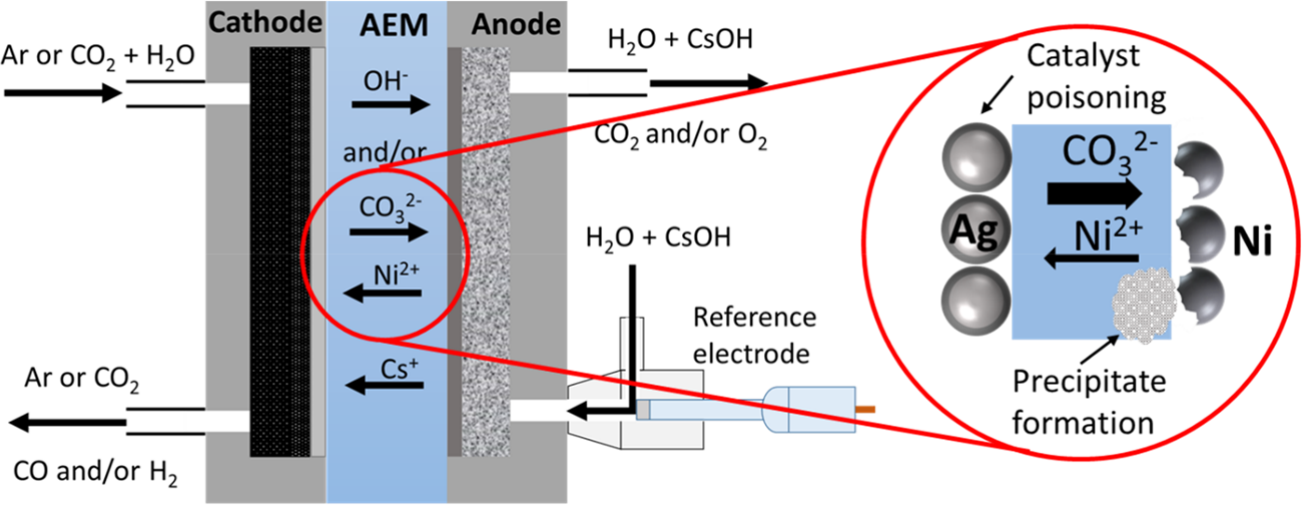
Schematic Representation of the Zero-Gap Electrolyzer Cell with a Possible Explanation of Catalyst Instability

**Figure 1:**
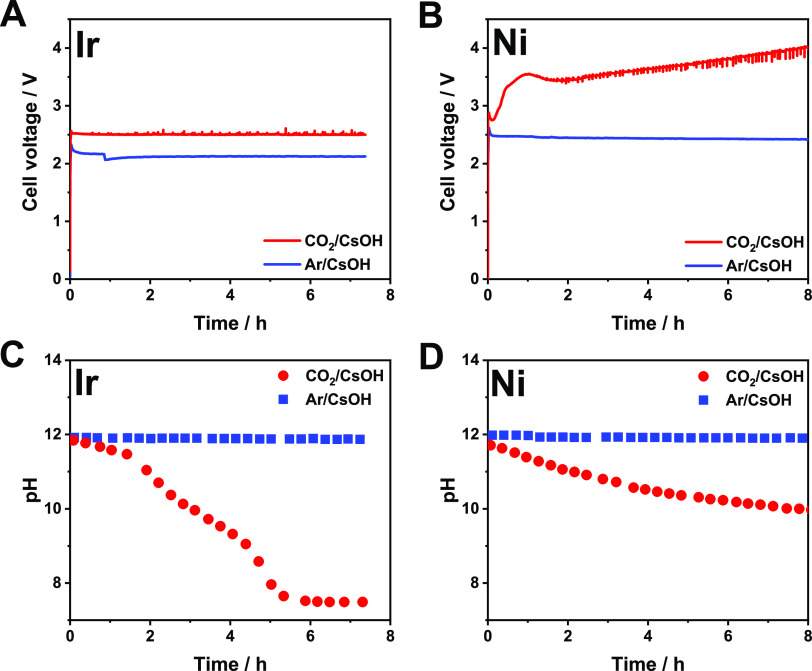
Chronovoltammetric curves recorded during continuous electrolysis using (A) Ir or (B) Ni anode catalyst. Changes in the anolyte pH during continuous electrolysis using (C) Ir or (D) Ni anode catalyst. Different cathodic gas feeds (Ar/CO_2_) were applied, as indicated in the figure legends. The electrolysis conditions were *T*_cathode_ = 60 °C, *j* = 100 mA cm^–2^, recirculated *V* = 1 dm^3^, 0.1 M CsOH anolyte, 12.5 cm^3^ cm^–2^ min^–1^ gas feed rate.

The operation of a custom-designed zero-gap electrolyzer cell (Scheme 1) was compared with Ir and Ni anode catalysts.^34,39,3^ First, the cathode was purged with humidified Ar gas, and a 0.1 M CsOH solution was recirculated in the anode compartment, hence performing water electrolysis in the electrolyzer cell as a baseline experiment. During constant current operation, the cell voltage was comparable when using Ir or Ni anodes, and it remained stable during the experiments in both cases (Figure 1A,B). Notably, in these cases, the transport of OH^–^ ions from the cathode to the anode maintains the ionic conduction between the electrodes. The stability of the system under HER conditions was also apparent from the stable H_2_ generation during long-term electrolysis experiments (Figure S1).

When a similar set of experiments was performed with cathodic CO_2_ feed, striking differences were found for Ir and Ni anode catalysts. A stable cell voltage (Figure 1A) and high CO formation Faradaic efficiency (FE_CO_) (Figure S2A) were recorded for the Ir catalyst. The pH of the recirculated anolyte decreased from 12 to 7.5 (*T*_anolyte_ ≈ 65 °C) within the time frame of the electrolysis (Figure 1C), caused by the transport of carbonate ions from the cathode to the anode. The rate of the pH decrease can be correlated with the amount of charge driven through the cell (detailed in the Supporting Information, section 2.1), which further confirms that carbonate ions are the dominant species participating in the ion conduction process under CO2RR conditions.

When the same experiments were performed using Ni anode catalyst, the initial cell voltage was similar to that measured with the Ir catalyst (2.7 V vs 2.5 V). Shortly after the beginning of the experiment, however, a voltage jump was observed, followed by a continuous increase in the cell voltage (Figure 1B). Notably, the cell voltage reached 3.5 V after 1 h and ∼4 V after 8 h of continuous operation. In parallel, the product formation rates for both H_2_ and CO decreased (Figure S2B), which will be discussed in what follows. To identify the reasons behind the high cell voltage, we recorded the anode potential during electrolysis by incorporating a reference electrode in the anode compartment (see Scheme 1). The measured anode potential values followed the same trend as the anolyte pH (Figure S3). Neither of these changes (pH, potential), however, are as significant for Ni as for Ir (Figure 1C,D and Figure S3A,B).

Electrochemical impedance spectroscopy (EIS) spectra recorded during CO_2_ electrolysis (applying Ni and Ir as anode catalysts) revealed further notable differences (Figures S4 and S5). The high-frequency intercept and the sum of the spans (“diameters”) of the arcs seen in the Nyquist representation of the EIS spectra for the electrolyzer cell with Ir anode remain similar, irrespective of the applied cathodic gas feed (CO_2_ or Ar). The same is true in the case of the Ni anode when only HER was performed on the cathode (i.e., Ar was fed to the cathode). However, as deduced from the semiquantitative fitting of the spectra, the series resistance and the total arc diameter (which is considered here as the total charge-transfer resistance) increased in parallel with the cell voltage when CO_2_ was fed to the cathode (Figures S6 and S7). The series resistance tripled (from 0.4 to 1.2 Ω cm^2^), while a ca. fourfold increase (from 3 to 12 Ω cm^2^) was measured in the total charge-transfer resistance. This indicates the deactivation of the catalyst(s) and/or changes in the catalyst/membrane interfacial resistances and increases the cell resistance.^41^

**Figure 2:**
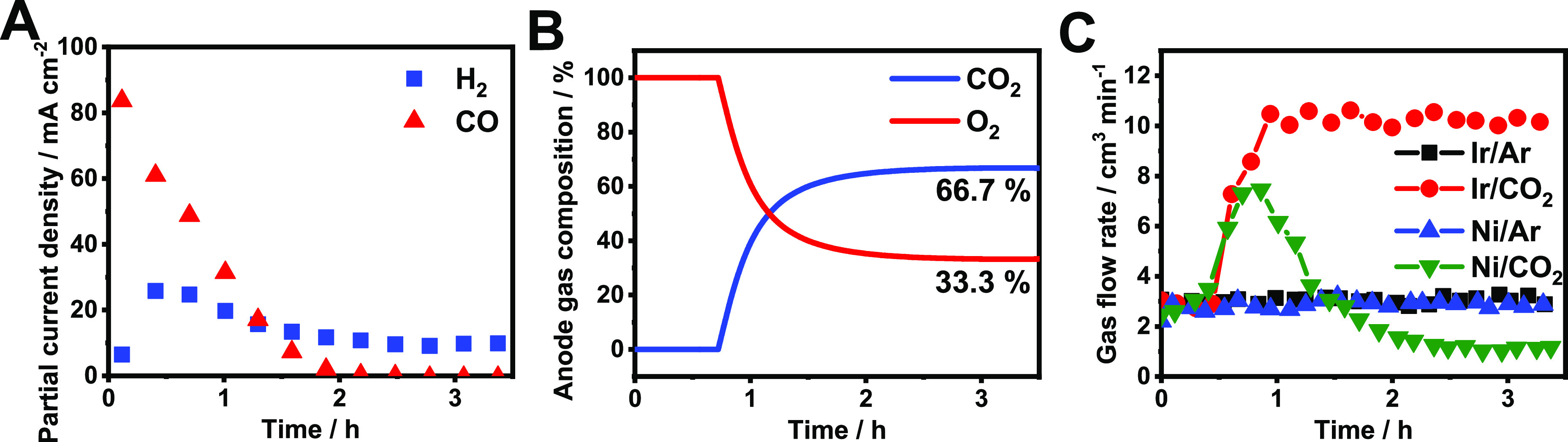
(A) Partial current densities of CO_2_ electrolysis products and (B) anode gas composition during continuous CO_2_ electrolysis using Ni anode catalyst. (C) Anode gas flow rate during continuous electrolysis using Ir or Ni anode catalyst applying Ar or CO_2_ cathodic feeds as indicated in the figure legends. The electrolysis conditions were 12.5 cm^3^ cm^–2^ min^–1^ gas feed on cathode, recirculated *V* = 100 cm^3^, 0.1 M CsOH anolyte, *T*_cathode_ = 60 °C, *j* = 100 mA cm^–2^.

During the CO_2_ electrolysis experiments with Ni anode catalyst, the partial current densities for both CO and H_2_ formation decreased rapidly (Figure 2A and Figure S2B). In fact, the total FE (∑FE) determined from the gas-phase products decreased continuously, and its typical value was 20–30% after 2 h of continuous electrolysis. Importantly, a negligible amount of liquid products was detected when the anolyte composition and the liquid collected from the cathode were analyzed by NMR spectroscopy (not shown here). This shows that part of the charge was consumed not in Faradaic reactions but in parallel parasitic process(es). This notion is further supported by the small change in the anolyte pH (a decay of only 2 pH units in 8 h, as opposed to the complete neutralization of the alkaline solution in less than 6 h when using Ir anode catalyst (Figure 1D)).

The anode gas composition (Figure 2B) and flow rate (Figure 2C) were also analyzed during these experiments. At the beginning of the electrolysis, pure oxygen was detected, which gradually changed to a 2:1 CO_2_:O_2_ mixture (a similar experiment with Ir is shown in Figure S8). This further confirms that carbonate ions are the dominant charge carriers between the electrodes.^3,39^ The anodically formed protons neutralize stoichiometric amounts of the alkaline anolyte, ultimately liberating CO_2_ once the pH becomes low enough (see further details in the Supporting Information, section 2.1). The gas flow rate is ∼3.2–3.4 cm^3^ min^–1^ when the cathode is fed with Ar gas using either Ir or Ni as anode (i.e., HER proceeds at the cathode), correlating with the value calculated from Faraday’s law (3.4 cm^3^ min^–1^). When the cathode feed was changed to CO_2_, a 3 times higher flow rate (∼10.0–10.5 cm^3^ min^–1^) was measured with the Ir anode after an initial period (as expected from the 2:1 CO_2_:O_2_ composition). A similar initial trend was witnessed when Ni was used as anode—the anode gas flow rate started increasing after a short initial period. After reaching a maximum, however, the gas flow rate started to decrease. The values confirm that part of the charge is not consumed in CO2RR (or HER) and OER; hence, less oxygen forms and less carbonate ions are transported through the membrane.

To exclude that the above-described phenomena are attributed only to the initial oxidation and dissolution of the Ni catalyst surface, the amount of charge required for the complete dissolution of the Ni catalyst was calculated (see Supporting Information, section 2.2), which would take less than a minute with the applied current.

## What Happens with the Ni Catalyst, and Where Is the Missing Charge?

**Figure 3:**
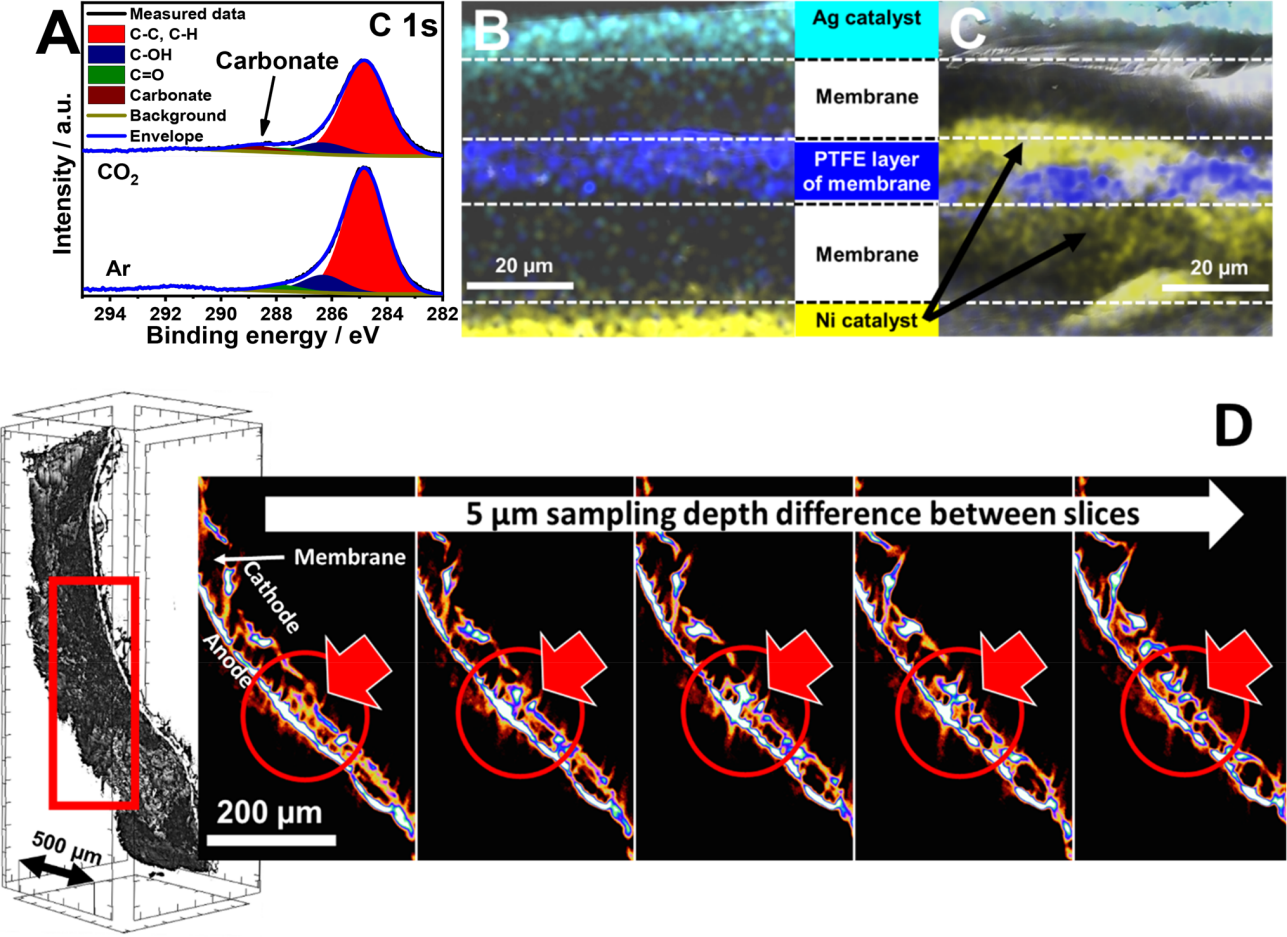
(A) C 1s region of the XPS spectra recorded for Ni anodes after electrolysis with CO_2_ or Ar feed on the cathode. Colored cross-section SEM-EDX images of the AEM before (B) and after (C) CO_2_ electrolysis with Ni anode, Ag cathode. Cyan, Ag; yellow, Ni; dark blue, F. (D) Micro-CT 3D reconstruction and side view images of the same AEM after CO_2_ electrolysis. The images present slices from different sample depths. The rectangle indicates the range from which the slices are displayed. The circle and arrow mark the place where Ni grows through the membrane.

The electrolyzer cells were disassembled after the electrolysis experiments, and all membrane electrode assembly (MEA) components were characterized to understand the changes leading to the high cell voltage and low ∑FE when using Ni as anode catalyst. X-ray photoelectron spectroscopy (XPS) measurements confirmed the formation of a Ni(OH)_2_/NiOOH layer on the anode catalyst surface when Ar gas was fed to the cathode (i.e., water-splitting was performed in the cell). More interestingly, we observed changes in the C 1s region when the Ar gas was switched to CO_2_ (i.e., CO2RR occurred) (Figure 3A). In this case, an additional carbon species at higher binding energies (288.8 eV) was necessary to fit the C 1s region, which corresponds to surface carbonate (see Figure S9).^42,43^ Based on the fitting and quantification of the XPS spectra, 40–50% of the surface Ni is in the form of NiCO_3_ (see Table S3 and further comments in the Supporting Information, section 2.6). Notably, Cs^+^ was also detected on the anode surface (Figure S10). Although this might also be carbonated (part of the detected surface carbonate might be in the form of Cs_2_CO_3_), the Cs amount cannot account for the increase of the surface carbonate by itself (as it is only ∼20% of the Ni). This change in the surface composition might contribute to both the increased series resistance and the increased charge-transfer resistance under the process conditions.

The AEM and the catalyst layers on it (transferred from the electrodes) were investigated before and after CO_2_ electrolysis, by taking cross-section scanning electron microscopy (SEM) images coupled with energy-dispersive X-ray (EDX) analysis (Figure 3B,C). Before electrolysis, the structure of the membrane was intact, with a compact PTFE layer in the middle and well-confined catalyst layers on the two sides of the membrane. This ordered structure changes drastically during the CO2RR experiments. The Ni layer is not confined to the anode side of the membrane anymore, but it appears inside the AEM as well, around the central Teflon reinforcement layer. Furthermore, at some points, it grows through the whole membrane. This phenomenon was further confirmed by micro-CT analysis, where Ni-containing plaques in the membrane were observed throughout the whole sample (Figure 3D). At some points, Ni fully penetrates through the membrane, bridging the two sides. We assume that the formation of these plaques is preferred in the microscopic cracks, pinholes, or other structural damages inherently present in the AEM.

These measurements indicate that Ni dissolves from the anode due to the locally acidic pH. The Ni^2+^ ions penetrate into the membrane, where precipitate forms because of the high local carbonate ion concentration and the low solubility of NiCO_3_. At some points, the precipitate grows across the AEM, thereby connecting the two catalyst layers. These local short-circuits might explain the low total ∑FE, as the charge driven through these high-resistance spots does not lead to product formation.

The cathode GDEs and cathode side of the membranes were investigated by XPS and SEM-EDX after CO_2_ electrolysis to see if Ni only enters the membrane or even passes through it. The XPS survey scans confirmed the presence of Ni in both cases: Ni(II) species were identified in the spectra recorded for the membrane and the GDE as well (Figure S11A,B). This was further confirmed by SEM-EDX measurements, where Ni was similarly detected on the cathode GDE after CO_2_ electrolysis with the Ni-coated anode (Figure S12). This means that a fraction of the dissolving Ni ions passes through the membrane and deposits on the cathode GDE. This leads to catalyst poisoning, explaining the decreasing CO2RR selectivity (Figure S2B), as HER is the preferred process on Ni.^44^ This process also contributes to the ∑FE decrease, as no products form in the dissolution–deposition of Ni (see calculations in the Supporting Information).

**Figure 4:**
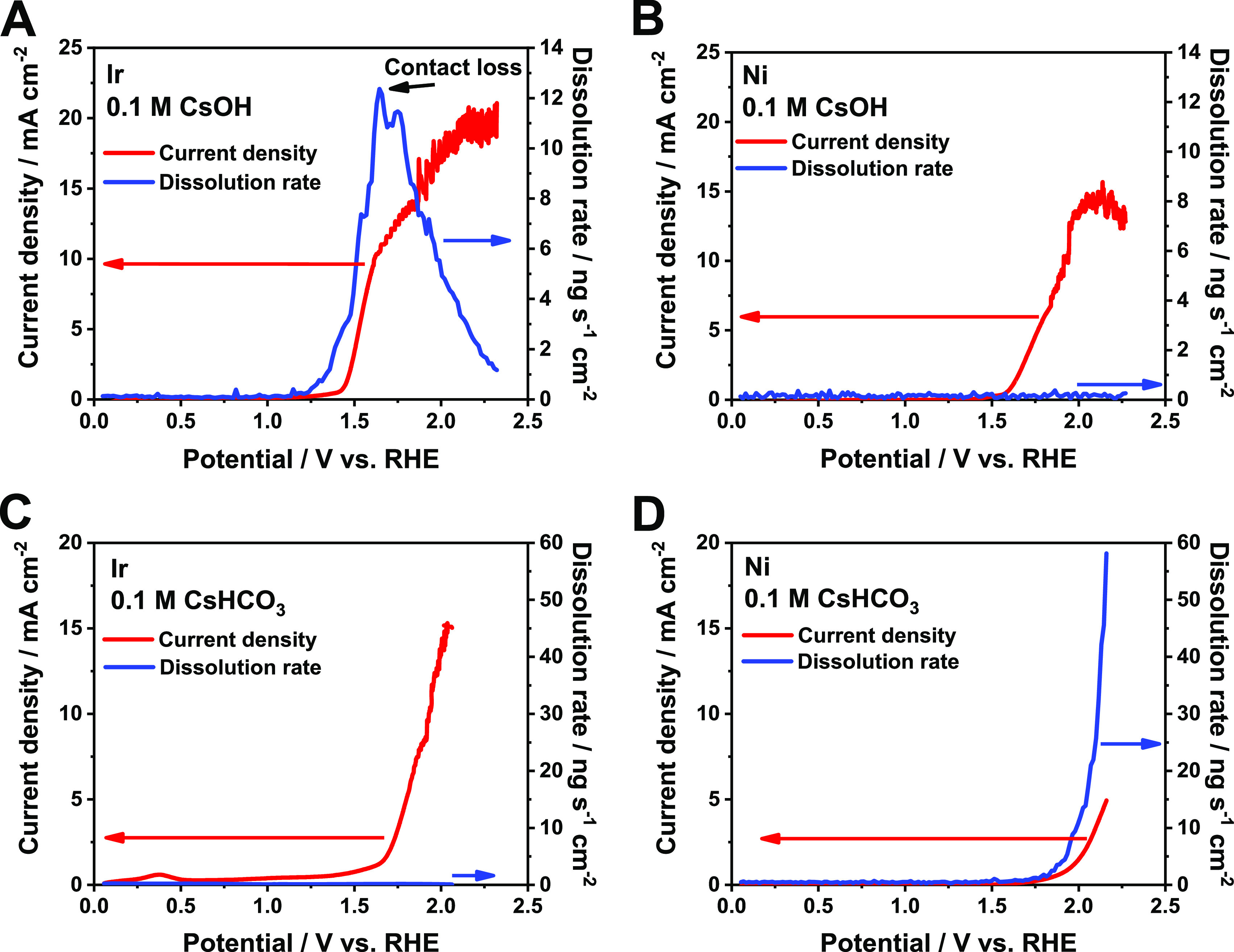
Online ICP-MS coupled linear sweep voltammetry measurements to study the stability of catalysts at room temperature, in different electrolytes: (A) Ir in 0.1 M CsOH, (B) Ni in 0.1 M CsOH, (C) Ir in 0.1 M CsHCO_3_, and (D) Ni in 0.1 M CsHCO_3_.

To directly probe the stability of Ni and Ir catalysts, electrochemical measurements in a three-electrode scanning flow electrochemical cell with online ICP-MS measurements were carried out both in alkaline media and under conditions which are closer to those under operation in a zero-gap flow cell (Figure 4).^4,45,46^ Noisy current, or even the current decrease with increasing potential, and also the contact loss can be observed in the figures due to bubbles forming on the surface of the examined catalyst in the scanning flow cell. In alkaline media (0.1 M CsOH), the dissolution of Ir starts at around 1.1 V vs RHE, in line with previous literature results.^4,9^ The rate of dissolution increases further upon increasing the potential, especially with the onset of OER at ∼1.5 V vs RHE (Figure 4A). Note that a similar potential was measured in the zero-gap cell with the Ir anode during HER and at the beginning of CO2RR (ca. 1.55 V vs RHE at *j* = 100 mA cm^–2^, Figure S3A). In the case of Ni in 0.1 M CsOH (Figure 4B), the current starts to increase above 1.55 V (vs RHE, OER onset), which is also in good correlation with the potential recorded in the zero-gap cell with Ni anode during HER and at the beginning of CO2RR (ca. 1.65 V at *j* = 100 mA cm^–2^, Figure S3B). However, no dissolution features can be observed in this case. In 0.1 M CsHCO_3_, the situation is reversed: no dissolution can be observed for Ir (Figure 4C), while notable Ni dissolution was seen (Figure 4D). The OER onsets shifted to more positive potential values, indicating that these catalysts are less active for OER in bicarbonate solution.

These measurements show that Ni is favored under alkaline conditions, while Ir is stable in near neutral medium, suggesting that Ni is a suitable anode catalyst in alkaline anolyte-operated, AEM-separated electrolyzer cells. However, in the case of zero-gap cell measurements, the *ions generated during electrolysis and passing through the membrane determine the pH conditions and not the bulk electrolyte solution*. Again, even if the initial anolyte pH is highly alkaline (e.g., pH 13 for 0.1 M CsOH at room temperature), it is neutralized during electrolysis, resulting in an almost neutral solution. This explains why Ir catalyst was found stable during prolonged electrolysis experiments and also suggests that the dissolution of Ni is unavoidable in AEM-separated zero-gap CO_2_ electrolyzer cells.

**Figure 5:**
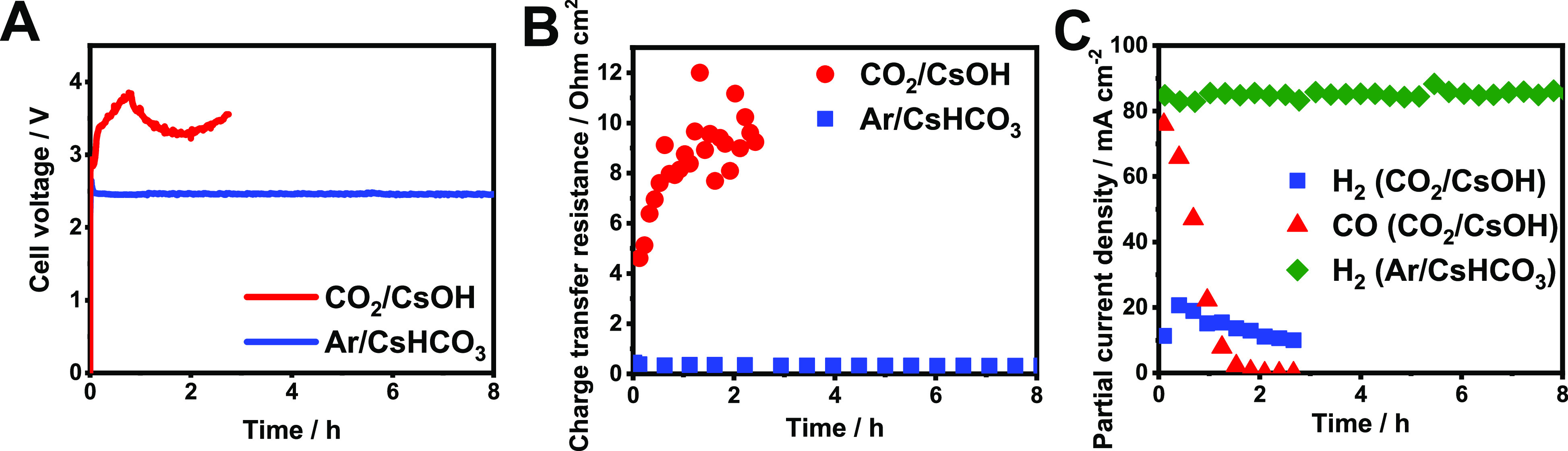
(A) Chronovoltammetric curves, (B) total charge-transfer resistances (derived from EIS measurements), and (C) partial current densities for CO and H_2_ formation during continuous electrolysis. Different cathodic gas feeds (Ar/CO_2_) and anolyte solutions (CsOH/CsHCO_3_) were applied, as indicated in the figure legends. The CsOH anolyte was non-recirculated, and fresh solution was continuously supplied to the anode. In the case of CsHCO_3_ anolyte, 1 dm^3^ was recirculated similarly to the previous measurements. The electrolysis conditions were Ni anode catalyst, *T*_cathode_ = 60 °C, *j* = 100 mA cm^–2^, and 12.5 cm^3^ cm^–2^ min^–1^ gas feed rate.

As mentioned above, the anode catalyst deactivation and the eventual cell failure may occur because of two reasons. The first is the anolyte pH decay, while the second is the local chemical environment of the anode catalyst, which in a zero-gap cell is determined by the ionic species crossing through the AEM. We have deconvoluted these effects by performing two sets of experiments (Figure 5): in the first, a near-neutral pH, 0.1 M CsHCO_3_ anolyte was applied (recirculated) to mimic the same conditions that developed during the CO_2_ electrolysis at the previous measurements, while humidified Ar was fed to the cathode. Under these conditions, the charge-conducting species through the AEM are OH^–^ ions. In this case, a stable cell performance was observed—the Ni anode did not fail (at least within the 8 h period of the experiment), even though the near-neutral pH of the anolyte could imply this. In the second experiment, the anolyte pH decrease was circumvented by continuously supplying fresh 0.1 M CsOH anolyte to the anode, without recirculation, while feeding the cathode with CO_2_—hence, carbonate ions maintain the conduction through the AEM. In this case, the cell voltage and the charge-transfer resistance of the cell increased rapidly (Figure 5A,B); meanwhile, the total FE decreased (Figure 5C) similarly to the results obtained using Ni anode catalyst with the recirculated 0.1 M CsOH anolyte (Figure 1B). These measurements prove that in zero-gap electrolyzer cells the ions crossing through the AEM are the most important in determining the activity and stability of the anode. The other fading mechanism—the dissolution of the anode catalyst caused by the pH decrease of the anolyte—might occur on a longer time scale, irrespective of the cell type (i.e., happens also in microfluidic cells with recirculated anolyte).

In conclusion, replacing the Ir anode catalyst with Ni in an AEM-separated zero-gap electrolyzer cell results in very high cell voltages during constant current CO_2_ electrolysis. This is accompanied by the decrease in CO2RR selectivity and the experimentally determined total FE. The reason behind this phenomenon is the dissolution of Ni under electrolysis conditions. This dissolution is a problem not only because of the catalyst loss but also because the Ni^2+^ ions penetrate into the membrane, where Ni(OH)_2_ and NiCO_3_ precipitates form. Furthermore, a fraction of the dissolved metal ions reaches the cathode, where they redeposit, poisoning the silver catalyst surface.

As also seen on the presented example of Ni, finding an alternative catalyst to replace Ir in CO_2_ electrolyzers is a grand challenge. Instead of searching for catalysts that are stable and active in alkaline water electrolysis, such candidates must be tested under conditions that are more relevant to CO_2_ electrolysis; namely, the optimal catalyst should bear excellent CO_3_^2–^ ion tolerance, and it should be stable and active at near-neutral pH.^47,48^ In the quest for novel anode catalysts, thermodynamic data on the stability of transition metals (i.e., Pourbaix diagrams) serve as a starting point.^9^ However, such data do not tell much about the stability of the given electrocatalyst in real conditions where factors such as electrolyte anions, temperature, and flow rate can have a dominant influence. Furthermore, real life electrolyzer cells operate far from equilibrium (e.g., high current density and large overpotential), which implies that kinetics becomes at least as important as thermodynamics in determining stability.

Both theoretical and experimental methods can assist catalyst screening. However, the proper test protocols can be only defined after testing a large number of electrocatalysts and drawing some initial conclusions on the kinetics and mechanism of catalyst degradation. The test protocols have to include screening the OER activity and stability of catalysts in near-neutral pH carbonate/bicarbonate solutions. These measurements shall be carried out on supported porous catalysts at high current density, mimicking the electrolyzer conditions. Online ICP-MS measurements offer an elegant way to correlate chemical and electrochemical data, enabling the rapid screening of potential catalysts.

## Supporting Information Available

Detailed experimental procedures, comments on the anolyte pH changes during electrolysis, further electrochemical measurements in zero-gap electrolyzer cells (anode potentials and partial current densities during CO_2_/water electrolysis, EIS results), and XPS and EDX analysis of the composition of the cell constituents before and after electrolysis (PDF)
