# Supplementary material for: Local Chemical Environment Governs Anode Processes in CO2 Electrolyzers
Source: ACS Energy Lett. 2021 Oct 7;6(11):3801–8. doi: 10.1021/acsenergylett.1c01937 (PMC8593866; doi:10.1021/acsenergylett.1c01937)
Supplement: Supplementary file 1 — nz1c01937_si_001.pdf [file nz1c01937_si_001.pdf]

– Supporting Information –

Local Chemical Environment Governs Anode Processes in CO_2 Electrolyzers

*Ádám Vass[1], Balázs Endrődi[1], Gergely Ferenc Samu[1], Ádám Balog[1], Attila Kormányos[1,2], Serhiy Cherevko[2], Csaba Janáky[1,]**

[1]Department of Physical Chemistry and Materials Science, Interdisciplinary Excellence Centre, University of Szeged, Aradi Square 1, Szeged, H-6720, Hungary

[2]Helmholtz-Institute Erlangen-Nürnberg for Renewable Energy (IEK-11), Forschungszentrum Jülich GmbH, Egerlandstr. 3, 91058 Erlangen, Germany

AUTHOR INFORMATION

Corresponding Author

*E-mail:janaky@chem.u-szeged.hu (C. Janáky).

Twitter: @JanakyLab

1. Experimental

1.1. Zero-gap electrolyzer flow cell

All experiments presented in this paper were performed in a custom-designed zero-gap electrolyzer cell with $A = 8\ cm^2$ active surface area, reported in our earlier publications.[1] Commercially available Ir black (PK catalyst, Fuel Cell Store) and Ni nanoparticles (<100 nm, Sigma-Aldrich) were applied as anode catalyst. The separator was Sustainion® X37-50 Grade RT anion exchange membrane (AEM, PTFE reinforced, thickness 50 µm, Dioxide Materials). The cathode gas diffusion electrode (GDE) was a silver nanoparticle (<100 nm, Sigma-Aldrich) coated carbon paper (Freudenberg H23C6) with a catalyst content of 1 mg cm^{-2}. The cathode GDE was prepared by spray coating the catalyst suspension on the gas diffusion layer. The suspension was made of the catalyst, distilled water, isopropanol and Sustainion XA-9 ionomer (Dioxide Materials). The anode electrodes were made also by spray coating a suspension of the catalyst powder on porous titanium frits with a catalyst content of 1 mg cm^{-2}. The membrane electrode assembly consisted of the cathode GDE, the AEM and the catalyst coated Ti frit anode.

The anolyte was 0.1 M CsOH or 0.1 M $CsHCO_3$ solution with a volume of 1000 cm^3 for the 8-10 h long and 100 cm^3 0.1 M CsOH for the 1-3 h long measurements. It was recirculated using a peristaltic pump with a flow rate of 100 cm^3/min and tempered in a water bath to keep

the cathode temperature at 60°C during the electrolysis. In the case of the measurement with continuous fresh anolyte fed ~16.5 dm^3 0.1 M CsOH was pumped through the anode. Humidified CO_2 (in case of CO_2 electrolysis, CO_2RR) or Ar (in case of hydrogen evolution reaction, HER) was fed to the cathode with 12.5 cm^3 cm^{-2} min^{-1} flow rate.

The cell was operated in galvanostatic mode (I = 0.8 A, j = 100 mA cm^{-2}) using a BioLogic VMP-300 potentiostat/galvanostat equipped with an impedance module and with a 5 A/10 V booster. The current densities were obtained by normalizing the current values to the geometric surface area of the electrodes ($j = I/A_{geometric}$).

The electrolysis products, CO and/or H_2, were monitored during the electrolysis using a Shimadzu GC-2010 Plus gas-chromatograph, equipped with a barrier discharge ionization (BID) detector. The cathode gas outlet was sampled in every ~20 minutes during the measurements. Faradaic efficiency of the CO_2 electrolysis was calculated from the GC results and the measured gas glow rate (Agilent ADM flow meter). In some experiments the product stream composition was simultaneously monitored using an m/z analyzer (SRS UGA200) equipped with an atmospheric sampling capillary.

The pH of the anolyte was also measured with the same sampling rate. A Mettler Toledo FiveEasy Plus FP20 pH meter was applied in automatic temperature compensation mode. The anolyte temperature was 65-70°C in all cases. A Hg/HgO reference electrode (in 1 M KOH, E^0=0.12 V vs. SHE) was integrated to the system connected to the cell at the anolyte inlet point.[2]

To get information about the changes of the catalysts, interfaces and the membrane during the electrolysis, electrochemical impedance spectroscopy (EIS) measurements were performed. EIS spectra have been taken at I = 0.8 A (j = 100 mA cm^{-2}) constant cell current with 10 mA perturbation amplitude, in the frequency range of 200 kHz to 1 Hz, with 10 points per frequency decade. The series resistance and the total charge transfer resistance (the sum of

the width of the two semicircles) were calculated by semi-quantitatively fitting the Nyquist representation of the EIS spectra using BioLogic EC-Lab software Z fit. A serial combination of two simplified Randles circuits was used to describe the measured EIS spectra. These fittings only served to determine the high frequency intercept (R_S), and the total width of the two arcs observed in the Nyquist plots. This latter is considered here as the total charge transfer resistance of the two electrode reactions.

The composition of anode gas outlet was analyzed with a BGA-244 type Binary Gas Analyser (Stanford Research Systems), to monitor the CO_2/O_2 ratio.[1] The anode gas outlet flow rate was measured using a bubble flowmeter.[3]

1.2. Scanning electron microscopy – Energy dispersive X-ray analysis

A Hitachi S-4700 scanning electron microscope (SEM) coupled with a Röntec EDX detector was used to take cross-section images of the cut GDEs. The microscope was operated at 10 kV acceleration voltage.

1.3. X-ray photoelectron spectroscopy

X-ray photoelectron spectroscopy (Al Kα) was carried out with a SPECS instrument equipped with a PHOIBOS 150 MCD 9 hemispherical analyzer. The analyzer was used in fixed analyzer transmission mode with 40 eV pass energy for the survey scans and 20 eV pass energy for the high-resolution scans. Charge referencing was done to the adventitious carbon (284.8 eV) on the surface of the sample. For spectrum evaluation, commercial CasaXPS software package was used.

1.4. X-ray micro-computed tomography analysis

A Bruker SkyScan 2211 Multiscale X-ray Nanotomograph (Bruker) instrument was used to record micro-CT images. The three-dimensional structure of the samples was scanned using an 11-megapixel CCD detector by applying a source voltage of 70 kV and current of 400 μA (in microfocus mode, with a resolution of 1 μm per pixel). NRecon reconstruction software

(Skyscan, Bruker) was used to reconstruct the projected images, while the CTAn and CTVox software (Skyscan, Bruker) were applied to carry out the image segmentation and visualizing the 3D-rendered objects, respectively.

1.5. Stability of anode catalysts at different pH values studied by on-line ICP-MS

The setup consists of a three-electrode electrochemical scanning flow cell (SFC) directly connected to an inductively coupled plasma mass spectrometer (ICP-MS, Perkin Elmer, Nexion 350X).[4–6] The measurements were carried out in 0.1 M CsOH (pH ~ 13) and in CO_2 saturated 0.1 M CsOH (pH=8.77, practically 0.1 M $CsHCO_3$) solutions for both catalysts.[7] 0.2 µl aliquots of a homogeneous suspension prepared from isopropanol, Milli-Q water, anion exchange ionomer (Sustainion XA-9), and the iridium or nickel catalyst powder by sonication via sonotrode and sonication bath was drop-casted on a glassy carbon electrode (SIGRADUR®, 5x5 cm). This resulted in a metal loading of 3.67 µg Ir and Ni per catalyst spot. After drying, this functioned as the working electrode. A glassy carbon rod served as the counter electrode. The current density values are related to the geometric surface area of the drop-casted catalysts. Ag/AgCl (in 3 M KCl) reference electrode was used to measure the potential. In order to avoid any possible chloride contamination from the reference electrode, the latter is connected to the outlet channel of the SFC via a capillary channel. All potential values are reported vs. the reversible hydrogen electrode (RHE), and were calculated by deducting −0.21 V vs. NHE for the Ag/AgCl reference electrode and 0.059 × pH values for the pH shift. The potential of the working electrode was held at 50 mV vs. RHE then a linear potential sweep was performed until contact loss due to vigorous bubble formation. ICP−MS calibration was performed daily by a four-point calibration slope made from standard solutions (Merck Centripur Ir, Ni, Re, Co) containing the investigated metal ions in a given concentration (0, 0.5, 1, 5 µg dm^{-3}) in either 0.1 M CsOH or in 0.1 M $CSHCO_3$. ^{187}Re and ^{59}Co served as internal standards. Delays

that arise from the transport of the electrolyte from the electrochemical flow cell to the ICP-MS were subtracted in order to directly correlate potential and dissolution data.

2. Results

2.1. Reason behind the anolyte pH change

Assuming that the ion conduction between the cathode and the anode is maintained by carbonate ions (as shown in earlier studies), for every 2 e^-, 1 CO_3^{2-} ion reaches the anolyte (equation (1) and (2)). This carbonate reacts with the H^+ ions formed during OER, and the forming H_2CO_3 reacts with CsOH, lowering the pH of the solution (equations (3-5)). All these combined results in a stoichiometry, where 2 e^- leads to the neutralization of 1 CsOH molecule to form $CsHCO_3$. Therefore, neutralizing a V = 1 dm^3, c = 0.1 M CsOH requires the transfer of n = 0.2 mol of electrons, which is Q = 19297 C charge. The cell current is I = 0.8 A, therefore the transfer of this charge takes 24120 s, about 6.7 hours. The time scales linearly with the anolyte volume; for a V = 100 cm^3 anolyte it is about 0.67 hours (40 minutes).

When working with Ir anode catalyst, the pH of the anolyte decreases in two steps, following the shape of a titration curve. The first step is related to the formation of Cs_2CO_3 and a CO_3^{2-}/HCO_3^- buffer. Subsequently, HCO_3^-/CO_2 systems forms, and as a consequence, CO_2 liberates from the solution.

$$CO_2 + 2e^- + H_2O \rightarrow CO + 2OH^- \quad (1)$$

$$2OH^- + CO_2 \rightarrow CO_3^{2-} + H_2O \quad (2)$$

$$H_2O \rightarrow 2e^- + {}^{1}/_{2}O_2 + 2H^+ \quad (3)$$

$$CO_3^{2-} + 2H^+ \rightarrow H_2O + CO_2 \quad (4)$$

$$CO_2 + OH^- \rightarrow HCO_3^- \quad (in\ 2\ steps) \quad (5)$$

Using Ir as anode catalyst, the experimentally determined times necessary to completely neutralize the alkaline anolyte are shorter than the calculated values (**Fig S8A**). The reason behind this is that in addition to carbonate conduction, CO_2 gas diffusion also occurs from the

cathode side to the anode side through the membrane, accelerating the process. When working with Ni anode catalyst the anolyte pH changes substantially slower, which is attributed to the loss of a large fraction of the total charge to a non-Faradaic process.

2.2. Charge associated with Ni dissolution

To identify whether Ni dissolution can consume a notable portion of the flown charge, we calculated the C charge necessary for the complete dissolution of the Ni catalyst:

- 1 mg cm^{-2} catalyst loading
- A = 8 cm^2 surface area
- m = 8 mg Ni catalyst
- n = 1.363 10^{-4} mol Ni catalyst

The total charge necessary for oxidizing Ni to Ni^{2+}: Q(2 e^-) = 2×F×n = 26 C

The total charge necessary for oxidizing Ni to Ni^{3+}: Q(3 e^-) = 3×F×n = 39 C

At the applied I = 0.8 A current these charges translate to 33 and 49 seconds of electrolysis, if all the charge is consumed in the dissolution.

2.3 pH of the anolyte

The shown pH values of the anolytes (**Fig 1. C, D**) might be confusing: pH 12 for 0.1 M CsOH and pH 7.5 for 0.1 M $CsHCO_3$. Notably, these values were recorded at 65-70°C. The automatic temperature compensation mode of the pH meter compensated the deviation resulting from the temperature constant of the Nernst equation. Further change of the pH at elevated temperature is caused by the change of the water ionization constant: T = 25°C $K_w = 1.008 \times 10^{-14}$, T = 75°C $K_w = 19.95 \times 10^{-14}$.[8] (T=60°C K_w= 9.55× 10^{-14}). The pH values

of the anolytes for each electrolysis were recorded at room temperature before and after the electrolysis (before heating up and after cooling down the anolyte, respectively) (see **Table S1, S2**).

Table S1. pH and temperature of the anolytes before and after electrolysis with Ir anode

	Ir					
	Ar/CsOH		CO_2/CsOH		Ar/$CsHCO_3$	
	T / ˚C	pH	T / ˚C	pH	T / ˚C	pH
Room temperature, before measurement	21.1	13	23.3	13	23.4	8.1
Heated, beginning of the measurement	66.5	11.9	67.6	11.8	67.7	8.3
Heated, end of the measurement	66.4	11.9	68.2	7.5	66.9	8.8
Room temperature, after measurement	23.7	13.1	23.4	8.1	24.3	9.1

Table S2. pH and temperature of the anolytes before and after electrolysis with Ni anode

	Ni					
	Ar/CsOH		CO_2/CsOH		Ar/$CsHCO_3$	
	T / ˚C	pH	T / ˚C	pH	T / ˚C	pH
Room temperature, before measurement	22.3	13.2	21.2	12.9	26.5	8.2
Heated, beginning of the measurement	66.2	12	66.6	11.7	64.8	8.5
Heated, end of the measurement	65.3	11.9	66.5	9.9	65.1	8.9
Room temperature, after measurement	22.4	13.1	22.9	10.2	22.5	9.1

2.4. Stability of the system during water splitting

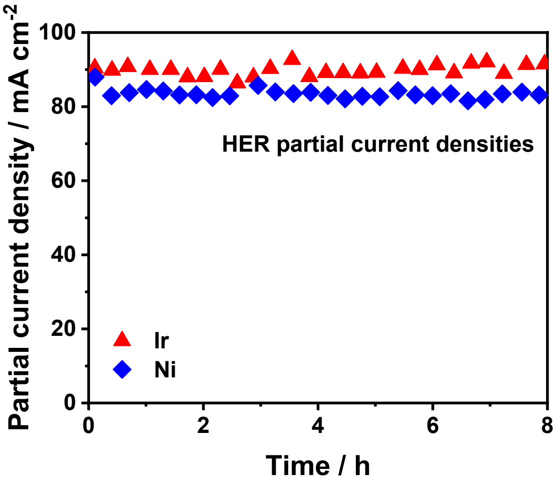

Figure S1. H_2 formation partial current densities during continuous water electrolysis using Ir or Ni anode catalysts. The electrolysis conditions were: 12.5 cm^3 cm^{-2} min^{-1} Ar feed on cathode, $T_{cathode}$ = 60 ˚C, j = 100 mA cm^{-2}, recirculated V = 1 dm^3 0.1 M CsOH anolyte.

2.5. CO_2 electrolysis in zero-gap flow cell

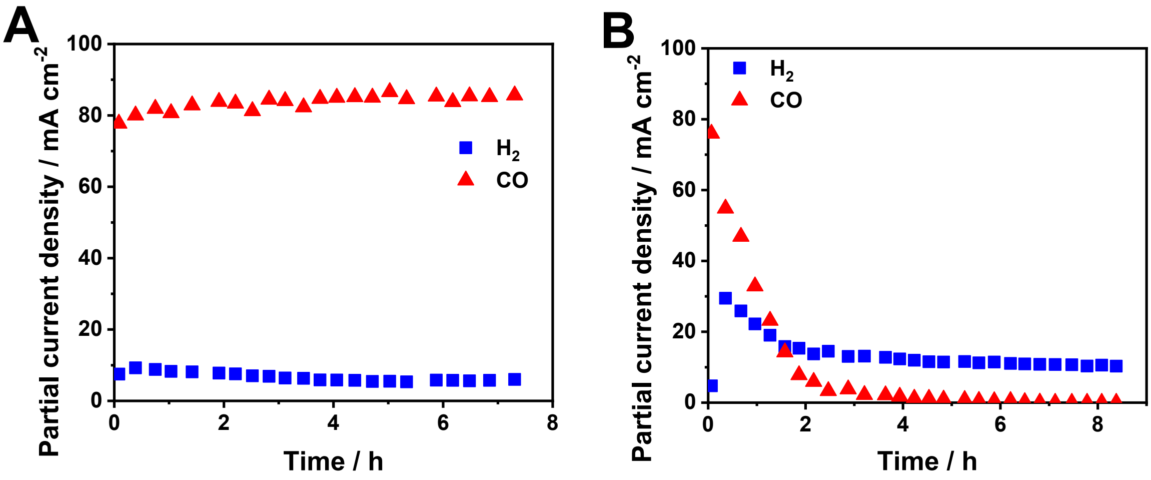

Figure S2. H_2 and CO formation partial current densities during continuous CO_2 electrolysis. (A) Ir anode, (B) Ni anode. The electrolysis conditions were: 12.5 $cm^3\ cm^{-2}\ min^{-1}$ CO_2 feed on cathode, $T_{cathode}$ = 60 ˚C, j = 100 mA cm^{-2}, recirculated V = 1 dm^3 0.1 M CsOH anolyte.

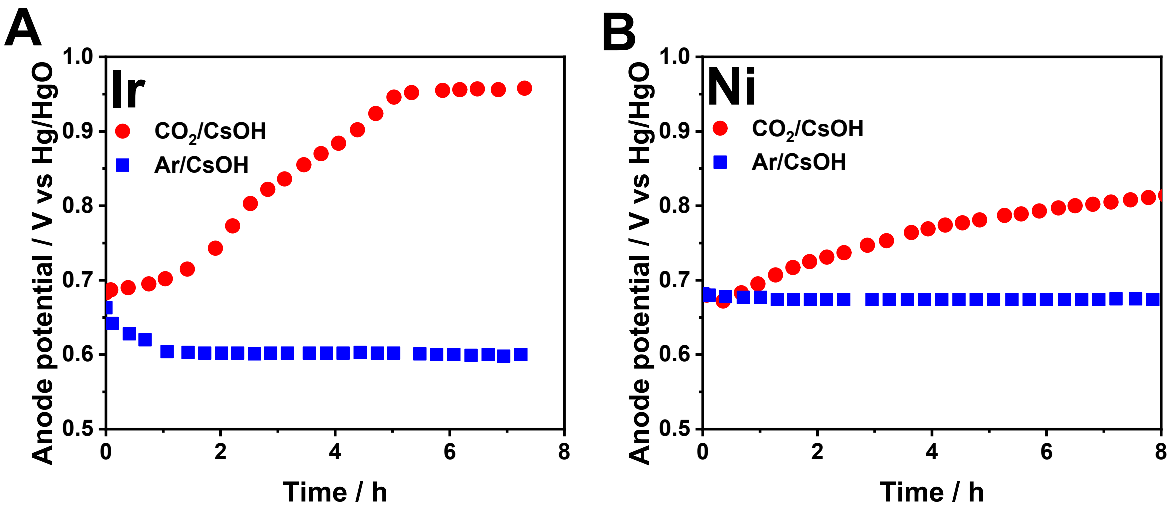

Figure S3. Anode potentials during continuous electrolysis using (A) Ir or (B) Ni anode catalysts. Different cathodic gas feeds were applied, as indicated in the figure captions. The electrolysis conditions were: 12.5 cm^3 cm^{-2} min^{-1} gas feed on cathode, $T_{cathode}$ = 60 ˚C, j = 100 mA cm^{-2}, recirculated V = 1 dm^3 0.1 M CsOH anolyte.

The potential values can be converted to the RHE scale based on the following equations, considering that the anolyte temperature is 65 ˚C (therefore the multiplier is not 0.059, but 0.067):

$$E(RHE) = E(Hg/HgO) + 0.067pH + E^0_{Hg/HgO}$$

$$E^0_{Hg/HgO} = 0.12V\ vs.\ SHE$$

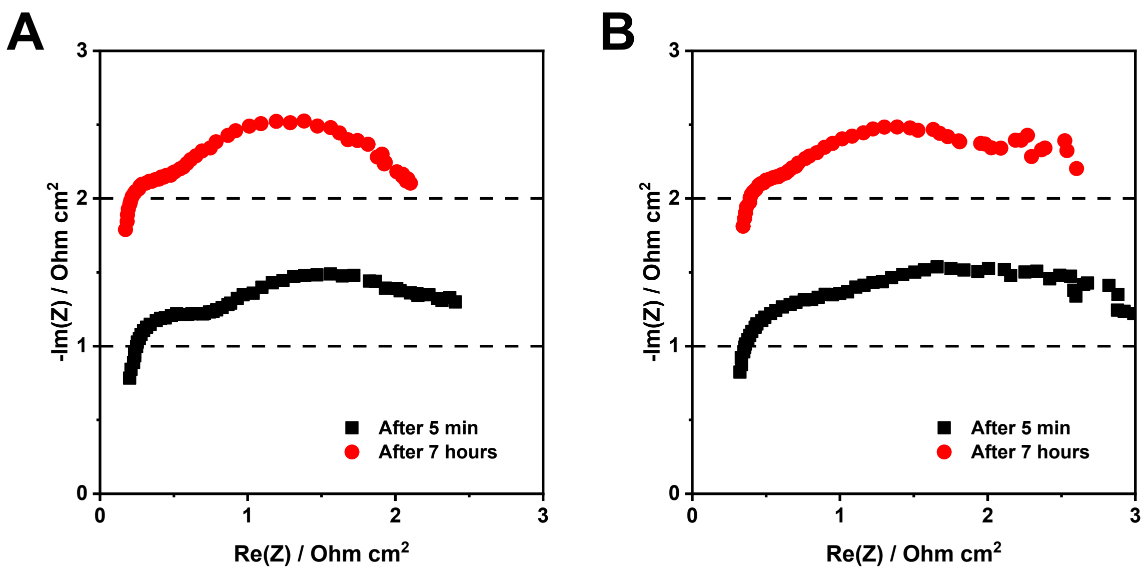

Figure S4. Electrochemical impedance spectra during continuous electrolysis using Ir anode catalysts. To help in distinguishing spectra they are shifted along the ordinate. Different cathodic gas feeds were applied: (A) Ar/CsOH, (B) CO_2/CsOH. The electrolysis conditions were: 12.5 cm^3 cm^{-2} min^{-1} gas feed on cathode, $T_{cathode}$ = 60 °C, j = 100 mA cm^{-2}, recirculated V = 1 dm^3 0.1 M CsOH anolyte.

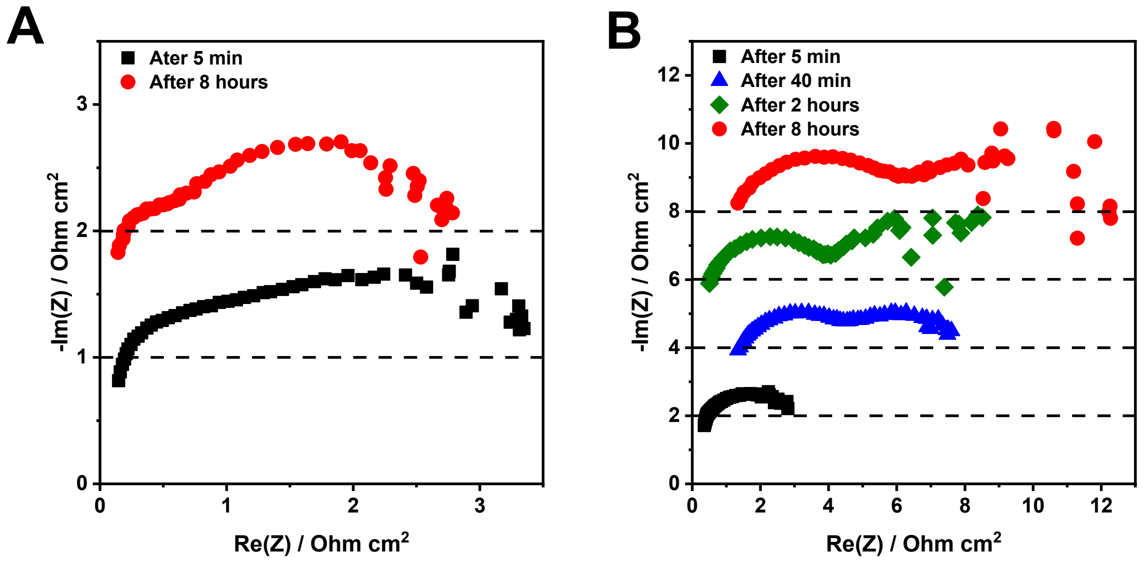

Figure S5. Electrochemical impedance spectra during continuous electrolysis using Ni anode catalysts. To help in distinguishing spectra they are shifted along the ordinate. Different cathodic gas feeds were applied: (A) Ar/CsOH, (B) CO_2/CsOH. The electrolysis conditions were: 12.5 cm^3 cm^{-2} min^{-1} gas feed on cathode, $T_{cathode}$ = 60 ˚C, j = 100 mA cm^{-2}, recirculated V = 1 dm^3 0.1 M CsOH anolyte.

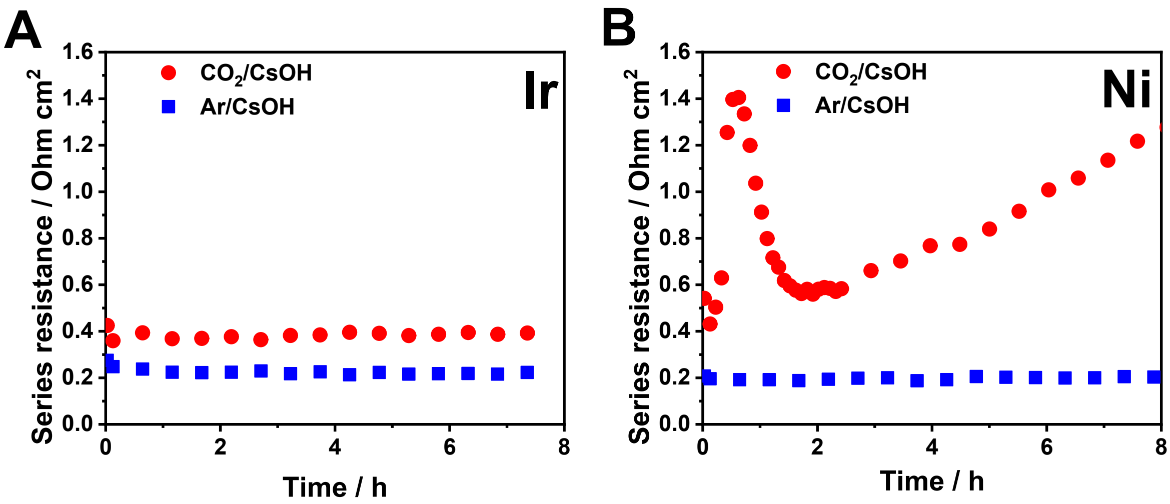

Figure S6. Series resistance of the cell during continuous electrolysis using (A) Ir or (B) Ni anode catalysts, derived from **Fig S4** and **S5**. Different cathodic gas feeds were applied, as indicated in the figure legends. The electrolysis conditions were: 12.5 cm^3 cm^{-2} min^{-1} gas feed on cathode, $T_{cathode}$=60˚C, j=100 mA cm^{-2}, recirculated V=1 dm^3 0.1 M CsOH anolyte.

An explanation for the increase in series resistance is that the initial increase can be attributed to the formation of surface $NiCO_3$ upon dissolution of Ni. As the process continues, more and more Ni^{2+} ion infuses into the membrane, forming $NiCO_3$ dendrites in it. After reaching a critical number (or length) of dendrites, percolation occurs, leading to decreased cell resistance.

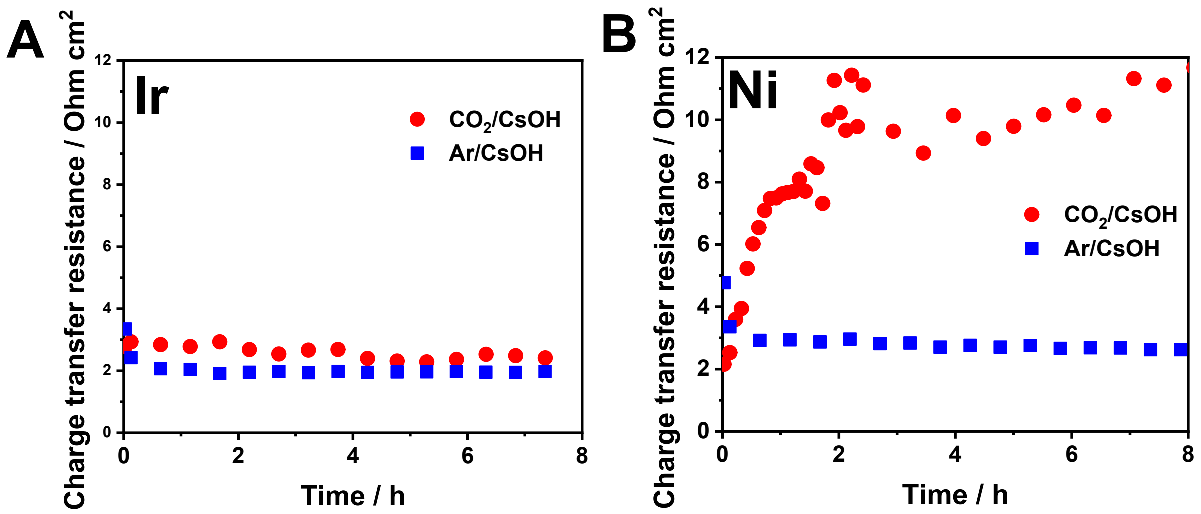

Figure S7. Total charge transfer resistance during continuous electrolysis using (A) Ir or (B) Ni anode catalysts, derived from **Fig S4** and **S5**. Different cathodic gas feeds were applied, as indicated in the figure legends. The electrolysis conditions were: 12.5 $cm^3\ cm^{-2}\ min^{-1}$ gas feed on cathode, $T_{cathode}$ = 60 ˚C, j = 100 mA cm^{-2}, recirculated V = 1 dm^3 0.1 M CsOH anolyte.

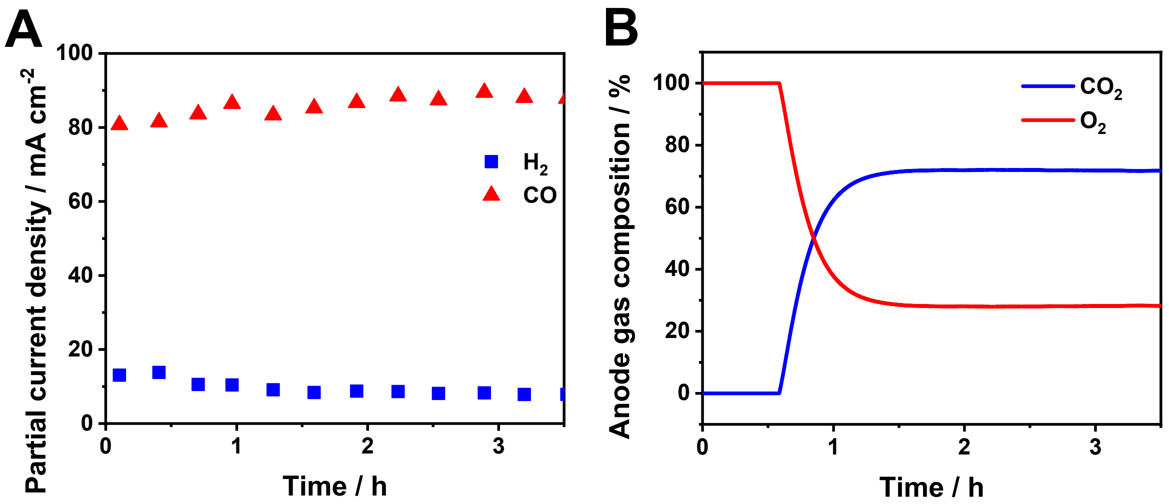

Figure S8. A) Partial current densities of CO_2 electrolysis products and **B)** anode gas composition during continuous CO_2 electrolysis using Ir anode catalyst. The electrolysis conditions were: 12.5 cm^3 cm^{-2} min^{-1} CO_2 feed on cathode, recirculated V = 100 cm^3 0.1 M CsOH anolyte, $T_{cathode}$ = 60 °C, j = 100 mA cm^{-2}.

2.6. X-ray photoelectron spectroscopy

As a reference commercially available $NiCO_3$ (98 % purity, Alfa Aesar) was also investigated (**Fig. S9**). Based on XPS measurements, the surface elemental composition of the two anode samples was determined (**Fig. S10**). The main difference is that the C 1s region of the sample after HER on the cathode did not have any contribution from surface carbonate species. In contrast, in the case of the anode where CO2RR was performed on the cathode side, significant carbonate contribution was observed (**Fig. 3A**). From this value the surface nickel carbonate content of the sample after CO2RR on the cathode can be estimated.

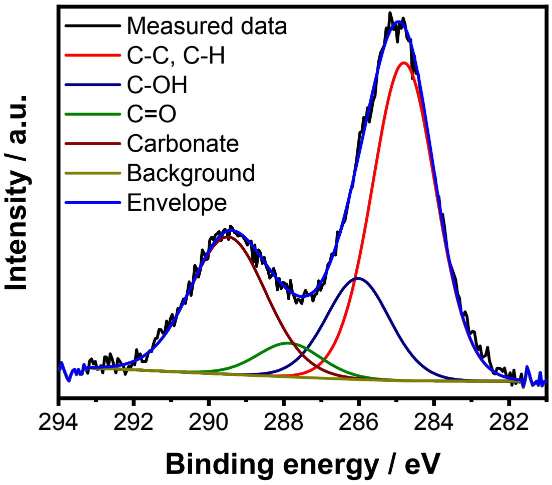

Figure S9. C 1s region of the XPS spectrum recorded for reference $NiCO_3$.

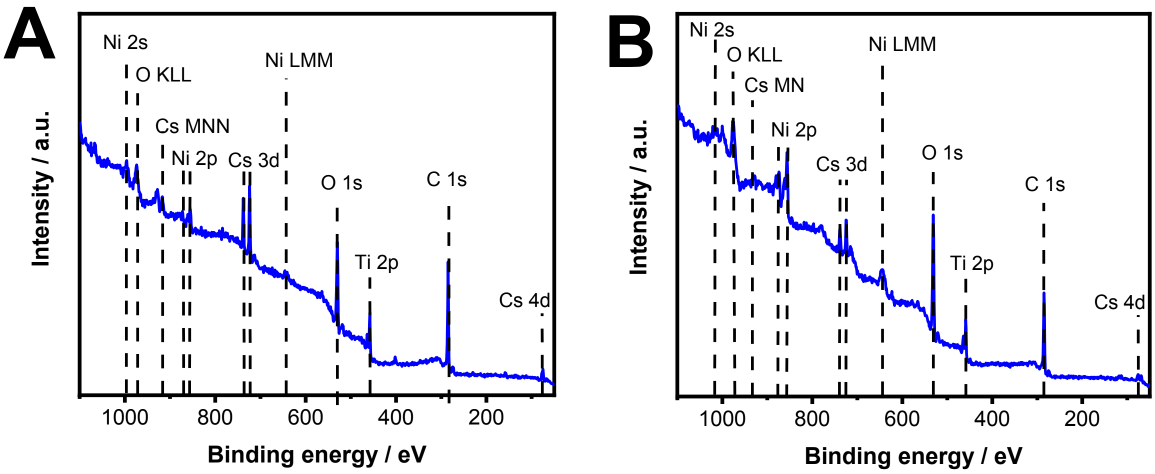

Figure S10. XPS survey scan of Ni anodes after electrolysis with (A) Ar or (B) CO_2 on the cathode, as shown in **Fig. 1B** in the main text.

Table S3. XPS C 1s region peak positions and contributions for Ni anode samples used in electrolyzer cells where HER or CO2RR was performed at the cathode, and that of $NiCO_3$ as a reference.

Sample	C-C, C-H peak		C-O peak		C=O peak		CO_3^{2-} peak	
	Pos.[a)] / eV	Contr.[b)] / %	Pos. / eV	Contr. / %	Pos. / eV	Contr. / %	Pos. / eV	Contr. / %
HER	284.8	84.7	286.3	11.6	287.8	3.7	-	-
CO2RR	284.8	82.4	286.3	9.7	287.8	3.5	288.8	4.4
$NiCO_3$	284.8	51.5	286	16.4	287.86	5.5	289.5	26.6

a) Position
b) Contribution

On the XPS survey scans of both the cathode GDE and the membrane (from the side facing the cathode) core lines related to Ni can be identified besides lines related to Ag (**Fig. S11**). This suggests that the nickel released from the anode side passes through the membrane and subsequently deposits on the cathode GDE.

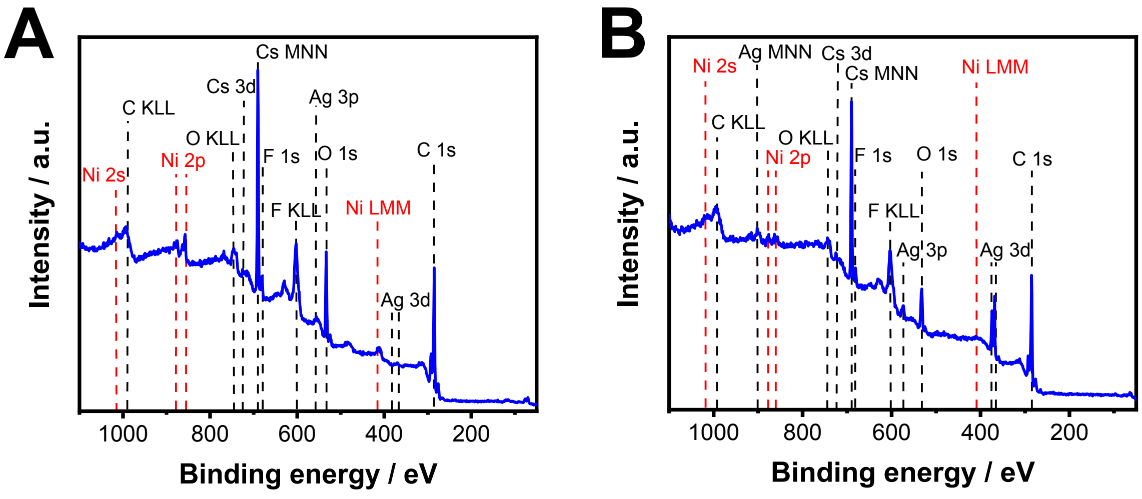

Figure S11. XPS survey scan of (A) the cathode side of the membrane and (B) the cathode GDE after CO_2 electrolysis with a Ni anode, as shown in **Fig. 1** in the main text.

Table S4. Quantification table of XPS measurements of the AEM cathode side and the cathode GDE after CO_2 electrolysis with new Ni anode, as shown in **Fig. 1** in the main text.

**Sample name**	**Ag / at%**	**Ni / at%**	**C / at%**	**F / at%**	**O / at %**
Membrane	0.1	2.2	59.1	25.1	13.4
GDE	2.4	0.9	63.9	24.2	8.5

2.7. Energy dispersive X-ray analysis

After CO_2 electrolysis employing Ni anode, the cathode GDE was investigated by SEM-EDX to see if Ni only enters the membrane or even passes through it and deposits at the cathode. Cathode GDEs after CO2RR with Ir anode and after HER with Ni anode were also investigated as comparisons. Ni appears only on the EDX spectrum of cathode GDE after CO2RR with Ni anode, as shown on Fig S13. In contrast, no anode catalyst was observed on the cathode in the other two reference cases.

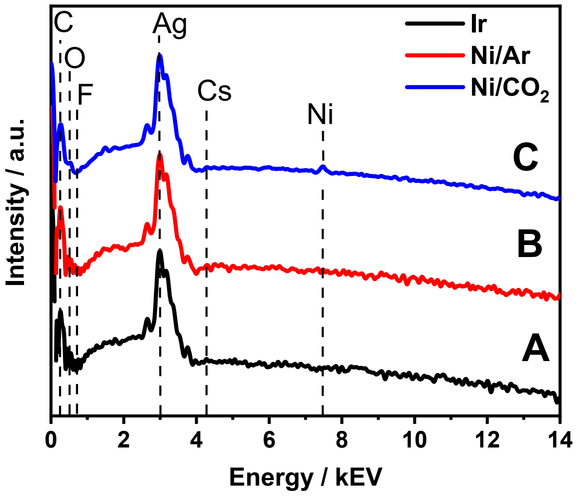

Figure S12. EDX spectra of the cathode GDE after (A) CO2RR with Ir anode, (B) CO2RR with Ni anode and (C) HER with Ni anode catalyst.

REFERENCES

(1) Endrődi, B.; Samu, A.; Kecsenovity, E.; Halmágyi, T.; Sebők, D.; Janáky, C. Operando Cathode Activation with Alkali Metal Cations for High Current Density Operation of Water-Fed Zero-Gap Carbon Dioxide Electrolysers. *Nat. Energy* **2021**, *6* (4), 439–448. https://doi.org/10.1038/s41560-021-00813-w.

(2) Salvatore, D.; Berlinguette, C. P. Voltage Matters When Reducing CO_2 in an Electrochemical Flow Cell. *ACS Energy Lett.* **2020**, *5* (1), 215–220. https://doi.org/10.1021/acsenergylett.9b02356.

(3) Larrazábal, G. O.; Ma, M.; Seger, B. A Comprehensive Approach to Investigate CO_2 Reduction Electrocatalysts at High Current Densities . *Accounts Mater. Res.* **2021**, *2* (4), 220–229. https://doi.org/10.1021/accountsmr.1c00004.

(4) Cherevko, S. Stability and Dissolution of Electrocatalysts: Building the Bridge between Model and "Real World" Systems. *Curr. Opin. Electrochem.* **2018**, *8*, 118–125. https://doi.org/10.1016/j.coelec.2018.03.034.

(5) Schalenbach, M.; Kasian, O.; Ledendecker, M.; Speck, F. D.; Mingers, A. M.; Mayrhofer, K. J. J.; Cherevko, S. The Electrochemical Dissolution of Noble Metals in Alkaline Media. *Electrocatalysis* **2018**, *9* (2), 153–161. https://doi.org/10.1007/s12678-017-0438-y.

(6) Kasian, O.; Geiger, S.; Mayrhofer, K. J. J.; Cherevko, S. Electrochemical On-Line ICP-MS in Electrocatalysis Research. *Chem. Rec.* **2019**, *19* (10), 2130–2142. https://doi.org/10.1002/tcr.201800162.

(7) Zhong, H.; Fujii, K.; Nakano, Y.; Jin, F. Effect of CO_2 Bubbling into Aqueous Solutions Used for Electrochemical Reduction of CO_2 for Energy Conversion and Storage. *J. Phys. Chem. C* **2015**, *119* (1), 55–61. https://doi.org/10.1021/jp509043h.

(8) Bandura, A. V.; Lvov, S. N. The Ionization Constant of Water over Wide Ranges of Temperature and Density. *J. Phys. Chem. Ref. Data* **2006**, *35* (1), 15–30. https://doi.org/10.1063/1.1928231.
